# Catamenial diabetic ketoacidosis in a rural area of Kananga, Democratic Republic of the Congo: a case report

**DOI:** 10.3389/fendo.2026.1935827

**Published:** 2026-09-25

**Authors:** John K. Bukasa, Branly K. Mbunga, Bedel Lukoki-Beudin, Guy Kikuni Makinga, Manasse Bukasa, Isis Kalambayi Kapinga, Claude Mbuyi, Daniel Lushimba, Eric Ditu Tshitshi, Patrick Ngoy Kalambayi, Ariel Makembi, Ayrton Bangolo

**Affiliations:** 1 Department of Endocrinology, University Hospital Center of Liege, University of Liege, Liege, Belgium; 2 Department of Nutrition, School of Public Health, University of Kinshasa, Kinshasa, Democratic Republic of Congo; 3 Nephrology unit, Department of Internal Medicine, University Clinics of Kinshasa, Kinshasa, Democratic Republic of Congo; 4 Community Health Service, Makala General Hospital, Kinshasa, Democratic Republic of Congo; 5 Department of Endocrinology, Manasse Endocrinology Clinics, Kananga, Democratic Republic of Congo; 6 Department of Internal Medicine, Notre Dame University of Kasayi, Kananga, Democratic Republic of Congo; 7 Medical Imaging and Diagnosis Unit, Nuclear Medicine Section, CGEA/CREN-K, Kinshasa, Democratic Republic of Congo; 8 University Hospital Center Of Guiana/Saint-Laurent, Cayenne, France; 9 Department of Hematology and oncology, John Theurer Cancer Center, Hackensack, NJ, United States

**Keywords:** catamenial, Democratic Republic of Congo, diabetic ketoacidosis, hormonal cycle, rural region

## Abstract

Diabetic ketoacidosis (DKA) is a life-threatening acute complication of diabetes mellitus most commonly triggered by infection, metabolic stress, or inadequate insulin therapy. We report an unusual case of a young woman with type 1 diabetes who experienced recurrent episodes of ketoacidosis during the premenstrual period, suggesting a catamenial form of DKA. This case highlights the importance of considering hormonal fluctuations related to the menstrual cycle in diabetes management and in identifying factors of imbalance. The patient presented with abdominal pain, severe fatigue, and polyuria-polydipsia, with a blood glucose level of 560 mg/dL, beta-hydroxybuturate of 3.6 mmol/L, potassium of 2.4 mmol/L and pH of 7.24. Inflammatory markers were absent and no usual precipitating factors were identified. Management included intravenous insulin therapy, rehydration, potassium supplementation, and transition to basal-bolus insulin. Part of the serum was sent to France for diabetes typing, confirming type 1 diabetes and additional assays. The patient reported that these episodes occurred 3 to 4 days before menstruation. This case underscores the need to consider hormonal fluctuations related to the menstrual cycle in diabetes management, particularly in a resource-limited settings, to adjust insulin strategies and prevent serious complications.

## Introduction

Diabetic ketoacidosis (DKA) is a metabolic emergency characterized by hyperglycemia above 2.5 g/L (250 mg/dL), metabolic acidosis with a pH < 7.3 or bicarbonate < 18 mmol/L, and ketonemia > 3 mmol/L (1–3). It is the most common hyperglycemic emergency and a leading cause of death among patients with diabetes (2–4). DKA typically occurs individuals with type 1 diabetes, resulting from absolute insulin deficiency. Its prevalence varies according to age, geography, income level, access to healthcare, and awareness of the disease (1, 5). It is frequently observed in children under six years of age, particularly in context of poverty, low income, and rural environments (3, 6). The mortality rate ranges from 2% to 5% in industrialized countries and may reach 24% in low-income countries (1, 4). This condition may mark the onset of type 1 diabetes or occur in previously diagnosed individuals following exposure to destabilizing factors. The most common precipitating factors included infectious stress, psychological stress, corticosteroid therapy, discontinuation of treatment, and intercurrent conditions such as stroke or myocardial infarction (7–9).

The literature reports a few cases of ketoacidosis associated with the menstrual cycle, a rare phenomenon known as catamenial, which typically occurs during the late luteal phase. This condition can trigger previously undiagnosed diabetes or contribute to ketoacidosis imbalance (10–15). The rarity of this case and its pathophysiological implications justify our decision to share it with the medical community. The patient lived in Kananga, a poor city in the Democratic Republic of Congo, with very limited healthcare resources for the diagnostic and treatment of diabetes. This situation was further aggravated by an inter-ethnic war in 2016. We encountered this patient during our teaching missions at the University of Kananga. We collected samples and transported part of the serum to Kinshasa and France for diabetes typing and additional assays; which confirmed type 1 diabetes.

## Patient information and clinical observation

A 17-year-old female patient, diagnosed with diabetes at age 15, presented with abdominal pain, severe physical fatigue, and polyuria-polydipsia syndrome. Her medical history indicated that diabetes had been diagnosed at age 15 in the context of severe dehydration. She also reported oligomenorrhea, with her last menstrual period occurring two months before the consultation. Additionally, she mentioned a probable case of type 2 diabetes in her mother and confirmed that her vaccination schedule was up to date.

The illness had begun three days before the consultation, with abdominal pain, breast tenderness indicating the onset of menstruation, and polyuria-polydipsia syndrome. She was taken to a clinic in the city of Kananga, where a blood glucose test showed a level of 560 mg/dL, and a urine dipstick test revealed ketone bodies without leukocytes or nitrites. The complete blood count was normal, showing no signs of inflammation.

## Diagnostic assessment

The lack of technical resources did not allow the measurement of pH, serum electrolytes, or glycated hemoglobin in the town of Kananga. Signs of severe dehydration were also observed, leading to the consideration of diabetic ketosis. No precipitating factors were identified.

We were consulted during our mission in this city and faced the realities on the ground, marked by the lack of technical resources. Blood gas analysis and serum electrolyte testing were unavailable, and human insulin was the only type accessible. During our discussions, the patient reported that these episodes often occurred 3–4 days before her menstruation. Due to the absence of laboratory facilities for biological assays in Kananga, we collected samples and sent them to Kinshasa for the measurement of pH, ketonemia, C-reactive protein (CRP), leukocytes, bicarbonates, and HbA1c. Part of the sample was sent to France for diabetes typing. These results are presented in **Table 1**.

**Table 1:** Available biological results and the place of testing.

Biological parameter	Results	Normal value	Location of examination
Blood glucose (g/L)	5.6 g/L	0.7-1 g/L	Kananga
Ketonemia (mmol/L)	3.6	< 0.6 mmol/L	Kinshasa
Ketonuria	+++	Negative	Kananga
pH	7.24	7.3-7.45	Kinshasa
C-reactive protein (mg/L)	6 mg/L	< 5 mg/L	Kinshasa
Leukocytes (/mm^3^)	10,000/mm3	4,000-10,000/mm^3^	Kananga
Bicarbonates (mmol/L)	12 mmol/L	>20 mmol/L	Kananga
Anti-GAD antibodies (IU/mL)	10 IU/mL	< 5 IU/mL	France
Anti-IA2 antibodies (IU/mL)	8 IU/mL	< 10 IU/mL	France
Anti-ZnT8 antibodies (U/mL)	18 U/mL	< 15 U/mL	France
HbA1c (%)	9.6%	< 7%	Kinshasa
Urine test strip	Negative	Negative	Kananga
Potassium (mmol/L)	2.4 mmol/L	3.5-5 mmol/L	Kananga
Thick blood	Negtive	Negative	Kananga

Isolated fasting glucose levels according to the phases of the menstrual cycle-follicular, ovulatory, and luteal, as shown in **Table 2**.

**Table 2:** Isolated glucose readings according to menstrual cycle phases.

Parameter	Follicular phase	Ovulatory phase	Luteal phase
Fasting blood glucose (g/L)	1.3 g/L	2.0 g/L	5.6 g/L
HbA1C (%)	8.5%	9.0%	9.6%

## Therapeutic intervention

An electric syringe was not available; therefore, we treated her with scheduled insulin injections, with 5 units administered intravenously per hour, along with rehydration and potassium supplementation until ketone bodies became negative. Subsequently, we transitioned to basal and rapid-acting insulin therapy. Since her type of diabetes was unknown, we naturally took the opportunity, with the consent of her parents, to collect samples that we transported to France for diabetes typing analysis.

## Follow up and outcomes

The patient has continues insulin therapy according to a basal-bolus regimen, administered regularly, with hemoglobin levels monitored every three months through the financial support of an NGO. She has been instructed to increase her basal insulin dose by 6 units and rapid-acting insulin doses by 3 units at the onset of menstruation, and to return to her usual doses at the end of the cycle. Follow-up evaluations are scheduled every three months at Kananga Provincial Hospital. She consulted a gynecologist, who prescribed an estrogen-progestin contraceptive to help regulate her menstrual cycles and mitigate hyperandrogenism.

## Discussion

This publication aims to inform the scientific community about catamenial ketoacidosis in the rural city of Kananga, Democratic Republic of the Congo.

This phenomenon was first described by Harrup and Mosenthal in 1918 at John Hopkins Hospital, followed by a second case in 1921, a third in 1932, and another in 1946 (15). Since then, numerous additional cases have been reported (9, 11, 15), and our case contributes to the existing body of literature. This is the first case diagnosed in Kananga and in the Democratic Republic of the Congo. It is likely that similar cases have remained undiagnosed, particularly given that the local medical community was unaware of the existence of this condition. The most frequent precipitating factors are psychological, traumatic, and infectious stress (2, 9, 16). Stress activates the sympathetic nervous system, which in turn stimulates the sympathetic ganglia to release acetylcholine. This neurotransmitter acts on the adrenal glands, inducing the secretion of catecholamines and cortisol, thereby promoting glycolysis and insulin resistance (17–19). It should also be noted that all the processes occurring during the luteal phase to prepare the endometrium for implantation of the fertilized ovum generate a certain low-grade inflammation, which underlies the production of proinflammatory cytokines capable of inducing insulin resistance (20, 21). The absence of infectious sites on physical examination and the absence of inflammatory markers in blood and urine, as observed in our patient, should prompt the clinician to investigate this phenomenon, particularly in women of reproductive age. It is important to report the decompensation or exacerbation of various pathologies during the premenstrual period, including gynecological, cardiovascular, dermatological, psychiatric, neurological, immunological, and other conditions. The slightly elevated CRP level of 6 mg/L was attributed to stress associated with ketoacidosis and premenstrual syndrome.

- Age at diabetes onset: most cases described worldwide presented this syndrome between 15 and 20 years of age (11, 15). Our patient fell within this usual range. We could not confirm whether it was of recent onset, particularly since the anamneses conducted prior to our consultation did not reveal it. It is therefore essential to allow the patient to express herself fully and not to neglect details provided during anamnesis. Physicians should actively investigate this phenomenon in the event of diabetic imbalance episodes in women of childbearing age. This syndrome, which constitutes one of the factors contributing to ketoacidotic decompensation, was not identified during the initial menstrual cycles in the cases described thus far. It was probably the underlying cause of decompensation, but clinicians overlooked it.

Several events occur in the pathophysiology of this condition.

Hormonal variations during the luteal phase of the menstrual cycle: this phenomenon arises in the late luteal phase, characterized by marked exposure to progesterone and, to a lesser extent, estrogens. These two hormones act as counter-regulatory agents, contributing to insulin resistance and increasing insulin requirements that are not adequately compensated before and during menstruation. This phase corresponds to significant hormonal changes that impair insulin sensitivity and promote lipolysis and ketogenesis, resulting in anti-insulin metabolic effects. Progesterone stimulates hepatic gluconeogenesis and lipolysis while reducing peripheral insulin sensitivity. Estrogens also contribute to insulin resistance and glucose intolerance.

- Peripheral insulin resistance: Under the combined influence of these two hormones, glucose uptake by peripheral tissues decreases, hepatic glucose production increases, and insulin levels rise by approximately 20%. If no therapeutic adjustment is implemented during this period, progressive hyperglycemia will ensue (22, 23).

Activation of lipolysis and ketogenesis: Relative insulin deficiency, combined with increased secretion of stress hormones (adrenaline and cortisol), promotes lipolysis, leading to the release of free fatty acids (FFAs) and their hepatic conversion into ketone bodies (β-hydroxybutyrate, acetoacetate, and acetone). If insulin deficiency worsens, ketone body production exceeds peripheral utilization, ultimately resulting in ketoacidosis (1).

- Low-grade stress: During the premenstrual period, several inflammatory processes occur in the endometrium, leading to low-grade stress that adversely affects cellular sensitivity to insulin action (24). Psychological and emotional stress must also be considered. The warning signs of menstruation, such as breast tension and abdominal pain, generate stress that stimulates the sympathetic nervous system (17), which in turn activates the sympathetic ganglia to release acetylcholine. This neurotransmitter acts on the adrenal glands, inducing the secretion of catecholamines and cortisol (25, 26), ultimately contributing to insulin resistance, glycolysis, and lipolysis.

- Additional factors such as anorexia during the premenstrual period and premenstrual vomiting reduced carbohydrate intake, thereby predisposing to fasting ketosis (27, 28). Premenstrual syndrome may also account for missed insulin injections. Particular attention should be paid to the irregularity of the menstrual cycle observed in this patient, which led us to suspect polycystic ovary syndrome (PCOS). PCOS is frequently associated with insulin resistance, and pelvic ultrasound confirmed the diagnosis (29–31). In this patient, a baseline of insulin resistance was already present, upon which hormonal variations and stress accumulated. The combination of these phenomena ultimately resulted in the progression from hyperglycemia to ketoacidosis.

In the presence of these phenomena, and in the absence of therapeutic adjustments, insulin deficiency leads to increased activation of adipocyte lipase, resulting in enhanced lipolysis. This process generates free fatty acids, which, once transported into the mitochondria of hepatocytes, are converted into ketone bodies (β-hydroxybutyrate, acetoacetate, and acetone). Although ketone bodies serve as an alternative energy source, their accumulation beyond a critical threshold induces systemic acidification, culminating in ketoacidosis (32).

From a therapeutic standpoint, the literature recommends appropriate patient education regarding the existence of this phenomenon, the warning signs of menstruation, and the need to increase insulin doses once premenstrual signs appear. In our patient, these signs included breast tenderness; however, the literature also describes pelvic pain, acne, and severe physical fatigue. Regular monitoring of blood glucose levels and testing for ketone bodies when blood glucose exceeds 2.5 g/L is advised. In the presence of digestive or respiratory manifestations suggestive of Kussmaul respiration, the patient should be referred to the emergency department for insulin therapy administered via an electric syringe, along with hydroelectrolytic correction. In our patient, who was treated with multiple daily insulin injections, therapeutic adjustments were made throughout the day to prevent ketoacidosis.

## Conclusion

This case highlights a rare but clinically significant form of catamenial diabetic ketoacidosis. It underscores the importance of an individualized approach to diabetes management in women, taking into account the hormonal specificities of the menstrual cycle. Recognition of this phenomenon enables the adaptation of insulin therapy and prevention of serious complications.

## Strengths and limitations

This presentation highlights a major strength in that it reports the first documented case of catamenial ketoacidosis in the Democratic Republic of the Congo, specifically in Kananga, one of the poorest cities in the country and a site of ethnic conflicts in 2017. This case illustrates the severe lack of technical resources, including the absence of an infusion pump for managing acute diabetes complications and the lack of a laboratory capable of measuring blood ion concentrations-an essential test in the management of ketoacidosis. This report serves as a call to action for the Société Francophone du Diabète (SFD) to provide material support to this city, located in one of the largest French-speaking countries in the world.

Weaknesses were noted, including the lack of essential biological and hormonal data, which would have considerably strengthened this case. Another limitation was that isolated glucose measurements were taken during each phases of the menstrual cycle instead of performing a comprehensive glycemic profile.

## Data Availability

The original contributions presented in the study are included in the article/supplementary material, further inquiries can be directed to the corresponding author/s.
